# Wirsungocele as a Rare Cause of Recurrent Pancreatitis: Etiology and Therapeutic Insights

**DOI:** 10.1002/deo2.70156

**Published:** 2025-06-05

**Authors:** Sorano Ichiya, Jun Nakahodo, Shunsuke Imaeda, Ryogo Minami, Fumio Kakizaki, Wataru Ujita, Hiroki Tabata, Kazuro Chiba, Toshiro Iizuka

**Affiliations:** ^1^ Department of Gastroenterology Tokyo Metropolitan Cancer and Infectious Diseases Center Komagome Hospital 3‐18‐22 Honkomagome Bunkyo‐Ku Tokyo 113‐8677 Japan

**Keywords:** endoscopic pancreatic sphincterotomy | magnetic resonance cholangiopancreatography | pancreatic duct stent | recurrent acute pancreatitis | wirsungocele

## Abstract

Wirsungocele, a cystic dilation at the end of the main pancreatic duct, is associated with recurrent acute pancreatitis. A 52‐year‐old man presented to our hospital with recurrent epigastric pain over an 8‐month period with a history of multiple medical visits for the same complaint. Endoscopic ultrasound (EUS) and magnetic resonance cholangiopancreatography (MRCP) revealed focal cystic dilatation at the end of the main pancreatic duct; thus, he was diagnosed with Wirsungocele. He underwent endoscopic pancreatic sphincterotomy and 5Fr 4 cm pancreatic duct stent placement; the pancreatic duct stent was removed 1 month later. Magnetic resonance imaging performed 3 months after discharge revealed no cystic dilation, and he has had no recurrence of pancreatitis for at least 6 months. Dysfunction of the sphincter of Oddi, weakening of the pancreatic duct wall, inflammation and recurrent stress, elevated intraductal pressure, and genetic and structural factors are suspected mechanisms behind the pathophysiology of Wirsungocele. Although the etiology of Wirsungocele is not known, its timely identification and treatment are critical to preventing recurrent episodes of pancreatitis. This case demonstrates the diagnostic value of combining MRCP and EUS as well as the therapeutic benefits of endoscopic intervention, including sphincterotomy and stent placement, in managing Wirsungocele‐associated recurrent pancreatitis. Given the paucity of reports on recurrent pancreatitis due to the Wirsungocele, we herein report this case and review the literature.

## 1 Introduction

Recurrent pancreatitis is defined as multiple attacks of acute pancreatitis that do not meet the diagnostic criteria for chronic pancreatitis. Notably, 30% of recurrent pancreatitis cases are considered idiopathic [1]; however, in some cases, recurrence and deterioration can be prevented by identifying the etiology. Wirsungocele, a rare cystic dilation of the terminal portion of the main pancreatic duct, has been identified as an etiology of recurrent pancreatitis. In this report, we describe a case of recurrent acute pancreatitis caused by a Wirsungocele, in which an endoscopic pancreatic sphincterotomy was performed and a pancreatic duct stent was inserted, resulting in a good outcome.

## 2 Case Report

**FIGURE 1:**
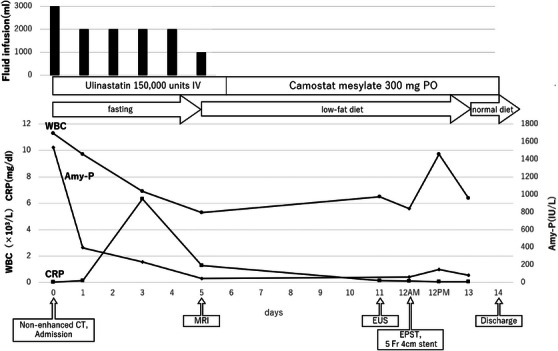
Clinical course of acute pancreatitis caused by the Wirsungocele. Blood tests at 12 PM were performed 2 h after the procedure to monitor for post‐endoscopic retrograde cholangiopancreatography (post‐ERCP) pancreatitis, and the transient elevation in white blood cell count (WBC) reflects this timing. IV: intravenous drip, PO: oral administration.

**FIGURE 2:**
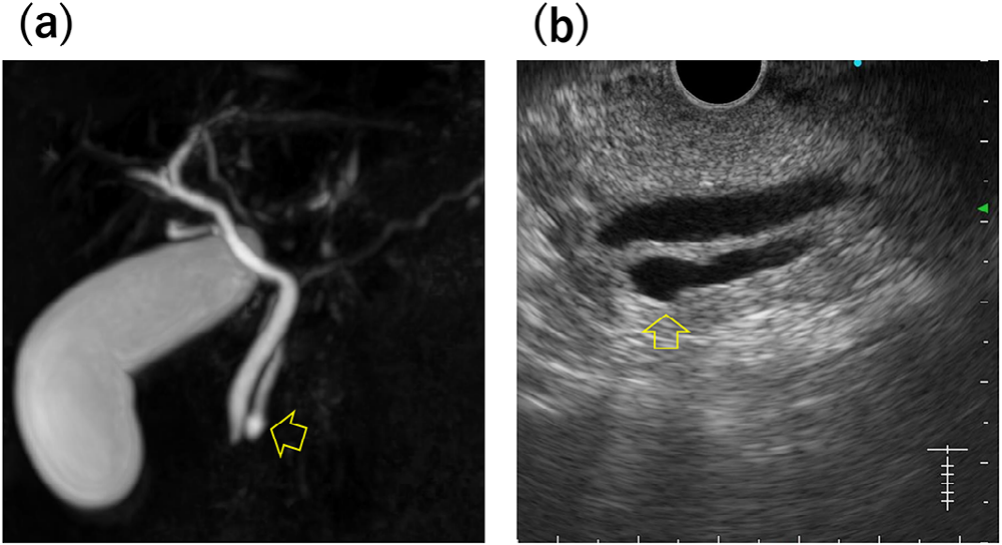
(a) Magnetic resonance cholangiopancreatography (MRCP) image reveals cystic dilatation of the main pancreatic duct above the papilla. (b) Endoscopic ultrasound (EUS) image shows focal cystic dilation of the pancreatic duct with a diameter of 5.7 mm at the pancreatic head. Arrows indicate the focal cystic dilatation area.

**FIGURE 3:**
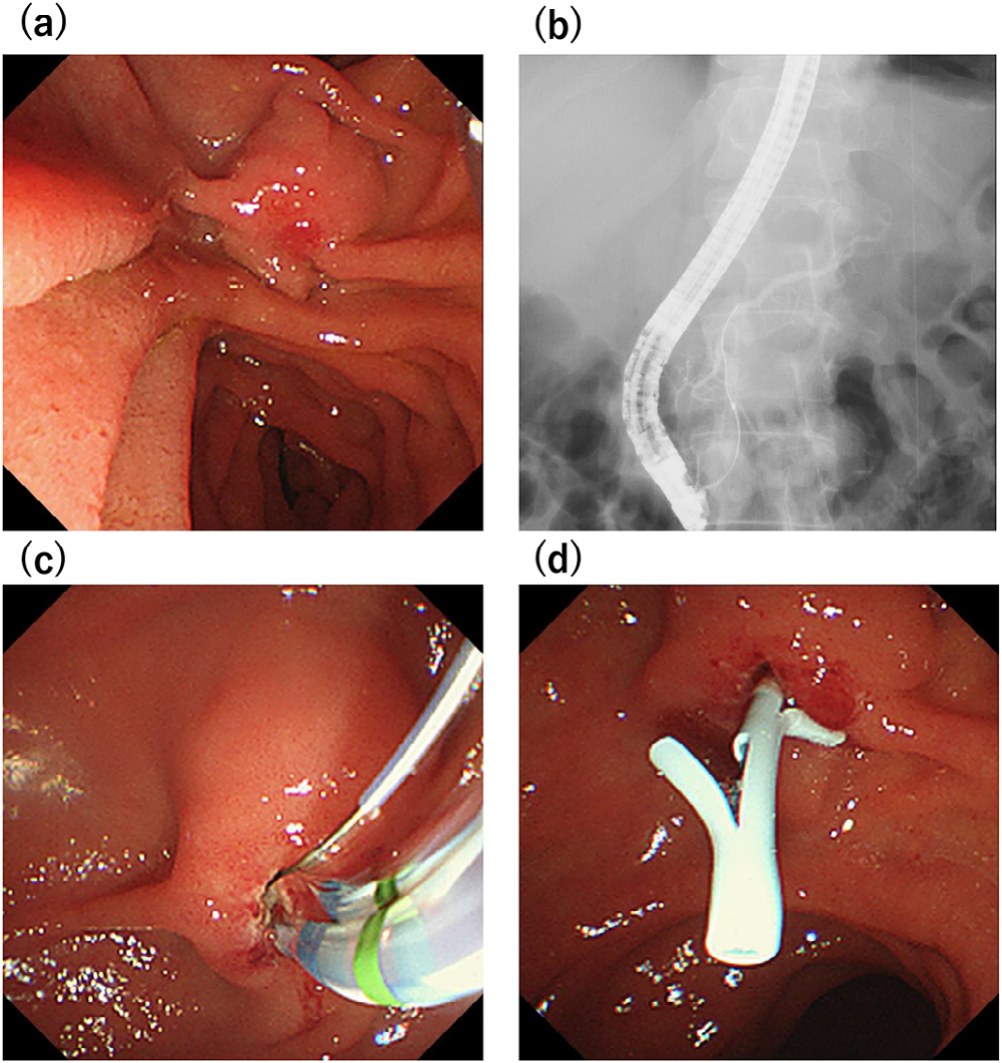
(a) We performed endoscopic retrograde cholangiopancreatography (ERCP) on the 12th day. The duodenal papilla is mildly erythematous. (b) There is no evidence of an incomplete pancreatic duct or pancreatic divisum. (c) An endoscopic sphincterotomy knife was inserted into the duodenal papilla, and an incision was made until the outflow of pancreatic juice was confirmed. (d) A 5Fr 4 cm single pancreatic stent was inserted.

The patient was a 52‐year‐old French Caucasian man with a chief complaint of epigastric pain that had recurred thrice in the past 8 months. Three weeks before presentation, he was evaluated elsewhere for elevated pancreatic enzymes, treated with camostat mesylate, and referred after magnetic resonance cholangiopancreatography (MRCP) revealed a Wirsungocele. He had a history of bronchial asthma and was on inhaled steroids. He consumed 12 g of alcohol per day on average until his visit to another hospital. Endoscopic ultrasound (EUS) at our hospital showed a focal cystic dilation of the main pancreatic duct without gallstones or pancreatic ductal stones. As alcohol‐induced acute pancreatitis could not be ruled out since he had consumed alcohol prior to the visit to another hospital, we decided to follow him up with careful observation. He had refrained from drinking alcohol since the previous pancreatitis; however, 2 days after the visit to our hospital, abdominal pain appeared and he was admitted to the emergency department of our hospital. On admission, physical examination revealed tenderness in the epigastric region. His body temperature was 36.9°C, pulse 106 bpm, and blood pressure was 155/101 mmHg. Blood tests on admission showed elevated white blood cell counts of 11,300/µL and Amy 1590 IU/L (Table 1). Due to his bronchial asthma, contrast was avoided, and non‐enhanced computed tomography (CT) was performed. The non‐enhanced CT indicated mild pancreatitis without main pancreatic duct dilatation. After admission, he was treated with fasting and fluids, intravenous protease inhibitors, and his white blood cell count, C‐reactive protein level, and Amy improved rapidly (Figure 1). Magnetic resonance imaging (MRI) performed on the sixth day of hospitalization showed a focal cystic dilatation of the end of the main pancreatic duct (Figure 2a). There is no evidence of an incomplete pancreatic duct or pancreatic divisum. EUS performed on the eleventh day of hospitalization revealed a focal cystic dilatation of the pancreatic duct at the pancreatic head with a diameter of 5.7 mm (Figure 2b). Radial EUS was initially performed, followed by convex EUS for longitudinal duct visualization; both revealed a maximum dilatation of 5.7 mm. We judged that the recurrent pancreatitis was caused by the Wirsungocele and terminated the procedure on the 12th day of hospitalization by performing endoscopic pancreatic sphincterotomy and placing a 5Fr 4 cm pancreatic duct stent (Figure 3). He had a good postoperative course and was discharged 2 days after the procedure. The pancreatic stent was removed 32 days after placement. EUS assessment at stent removal was limited by the stent, hindering the evaluation of focal cystic dilatation for the main pancreatic duct. MRCP image obtained on day 105 after discharge, which corresponds to day 75 after pancreatic stent removal: the previously noted focal cystic dilation of the pancreatic duct had resolved (Figure S1).

**TABLE 1:** Blood test results on admission to our hospital.

WBC	11300	/µL	LDH	228	IU/L
RBC	447	×10^3^/µL	**T‐bil**	0.9	mg/dL
Hb	13.6	g/dL	**D‐bil**	0.4	mg/dL
Ht	39.3	%	**Amy**	1590	IU/L
Plt	21.2	×10^4^/µL	**Amy‐P**	1535	IU/L
			**BUN**	14	mg/dL
TP	6.8	g/dL	**Cre**	0.82	mg/dL
Alb	4.5	g/dL	**Glu**	190	mg/dL
AST	71	U/L	**Na**	140	mmol/L
ALT	92	U/L	**K**	3.9	mmol/L
ALP	83	U/L	**Cl**	145	mmol/L
γ‐GTP	287	U/L	**Ca**	8.9	mg/dL
CK	145	U/L	**CRP**	<0.03	mg/dL

Abbreviations: WBC, White Blood Cell Count; RBC, Red Blood Cell Count; Hb, Hemoglobin; Ht, Hematocrit; Plt, Platelet count; LDH, Lactate Dehydrogenase; T‐bil, Total Bilirubin; D‐bil, Direct Bilirubin; Amy, Amylase; Amy‐P, Pancreatic Amylase; BUN, Blood Urea Nitrogen; Cre, Creatinine; TP, Total Protein; Alb, Albumin; Glu, Glucose; AST, Aspartate Aminotransferase; ALT, Alanine Aminotransferase; ALP, Alkaline Phosphatase; γ‐GTP,γ‐Glutamyl Transpeptidase; CK, Creatine Kinase; Na, Sodium; K, Potassium; Cl, Chloride; Ca, Calcium; CRP, C‐Reactive Protein.

## 3 Discussion

Cystic dilatations of the dorsal or ventral pancreatic ducts are referred to as Santorinicele or Wirsungocele, respectively. Unlike conditions defined by specific duct diameters, these diseases are characterized by localized cystic morphology and relative dilation, as pancreatic duct diameters naturally fluctuate with age. The first description of the Wirsungocele appeared in 2004 [2], and its association with recurrent pancreatitis was documented in 2008 [3].

MRCP has been established as a noninvasive and highly accurate diagnostic tool for Wirsungocele. Evrimler et al. [4] highlighted the efficacy of MRCP for diagnosing Wirsungocele, and Zhao et al. [5] further emphasized its value in understanding the etiopathogenesis of recurrent pancreatitis. Complementary to MRCP, EUS has also demonstrated significant diagnostic utility. Dhir et al. [6] and Mezzina et al. [7] reported that EUS plays a critical role in the precise evaluation and management of a Wirsungocele. In the present case, the combination of EUS and MRCP proved instrumental in accurately diagnosing the condition and formulating a treatment plan.

The pathophysiology of the Wirsungocele is being elucidated, with five primary mechanisms proposed in the literature: 1. Dysfunction of the Sphincter of Oddi: Uncoordinated movements due to abnormal autonomic control impair pancreatic juice drainage, leading to increased ductal pressure and subsequent cyst formation [2]. 2. Weakening of the Pancreatic Duct Wall: Dysfunction of the sphincter of Oddi or repeated inflammatory episodes may weaken the wall of the pancreatic duct, predisposing it to cystic dilatation [4]. 3. Impaired Pancreatic Drainage and Elevated Intraductal Pressure: Obstruction of the distal pancreatic duct results in increased pressure within the duct, promoting cyst formation [5]. Although pancreas divisum was absent in this case, Wirsungocele‐associated outflow impairment may have elevated ductal pressure, triggering pancreatitis. 4. Inflammation and Recurrent Stress: Chronic or acute pancreatitis induces damage to the ductal wall, facilitating cystic changes [8]. 5. Genetic and Structural factors: Genetic or structural anomalies, such as pancreas divisum, may also contribute to Wirsungocele development [8]. Genetic testing was not performed, but a functional abnormality was considered likely given the absent family history. As most Wirsungocele cases are Western, the patient's Caucasian background suggests possible racial differences. Although a direct causal relationship between the Wirsungocele and acute pancreatitis remains unproven, Gonoi et al. [9] identified a statistically significant association between the Wirsungocele and chronic asymptomatic pancreatic enzymuria, suggesting that abnormal duct morphology plays a significant role in the pathophysiology of pancreatitis. Additionally, Evrimler et al. [4] hypothesized that the size of the Wirsungocele correlates with the frequency of recurrent pancreatitis, highlighting the need for individualized treatment strategies based on cyst morphology and size.

Treatment Strategies: Endoscopic treatment has emerged as a viable option for managing Wirsungocele‐associated recurrent pancreatitis. Gupta et al. and Mezzina et al. [3, 7] reported success with endoscopic sphincterotomy (EST) and pancreatic duct stenting, typically with stent placement for a month. Cheung et al. [8] described a case involving a 7‐year‐old boy with three episodes of acute pancreatitis over 2 months. Following endoscopic pancreatic sphincterotomy and 6‐mm balloon dilation, a 5‐Fr pancreatic duct stent was inserted and subsequently removed after 4 weeks. All six previously reported cases with described treatment strategies underwent EST; pancreatic stent placement for four weeks was implemented in three of the cases, whereas dilation with a 6‐mm balloon was performed in one case. However, the clinical significance and the therapeutic efficacy of balloon dilation remain unclear. In this case, adequate drainage was achieved with EST and stent placement alone, rendering balloon dilation unnecessary.

In the present case, a combination of imaging modalities—MRCP and EUS—and endoscopic intervention proved effective for diagnosis and treatment. This integrated approach not only facilitated the identification of the Wirsungocele but also enabled patient‐tailored management strategies aimed at mitigating recurrent pancreatitis.

Diagnosing and treating Wirsungocele requires a multifaceted approach involving imaging and endoscopic interventions. MRCP and EUS complement each other in providing detailed anatomical and functional insights, while endoscopic procedures address the underlying mechanical and structural abnormalities. High‐resolution EUS allowed detailed lesion assessment and exclusion of coexisting lesions, while MRCP identified cystic dilatation suggestive of Wirsungocele and proved valuable for both diagnosis and post‐treatment evaluation of the pancreatic duct, aiding assessment of therapeutic efficacy. Further accumulation and analysis of clinical cases will contribute significantly to advancing our understanding of the Wirsungocele, optimizing treatment protocols, and improving patient outcomes.

## Conflicts of Interest

The authors declare no conflicts of interest.

## Ethics Statement

The authors report the details of the patient's case in accordance with the ethical standards of the Declaration of Helsinki.

## Consent

Written informed consent was obtained from the patient for the publication of this case report and its accompanying images.

## Clinical Trial Registration

Not applicable.

## Supporting information



MRCP image obtained on day 105 after discharge, which corresponds to day 75 after pancreatic stent removal: the previously noted focal cystic dilation of the pancreatic duct had resolved.

Video of the Endoscopy
