# Supplementary figures and images for: Wirsungocele as a Rare Cause of Recurrent Pancreatitis: Etiology and Therapeutic Insights
Source: DEN Open. 2025 Jun 5;6(1):e70156. doi: 10.1002/deo2.70156 (PMC12140840; doi:10.1002/deo2.70156)

## Slide 1
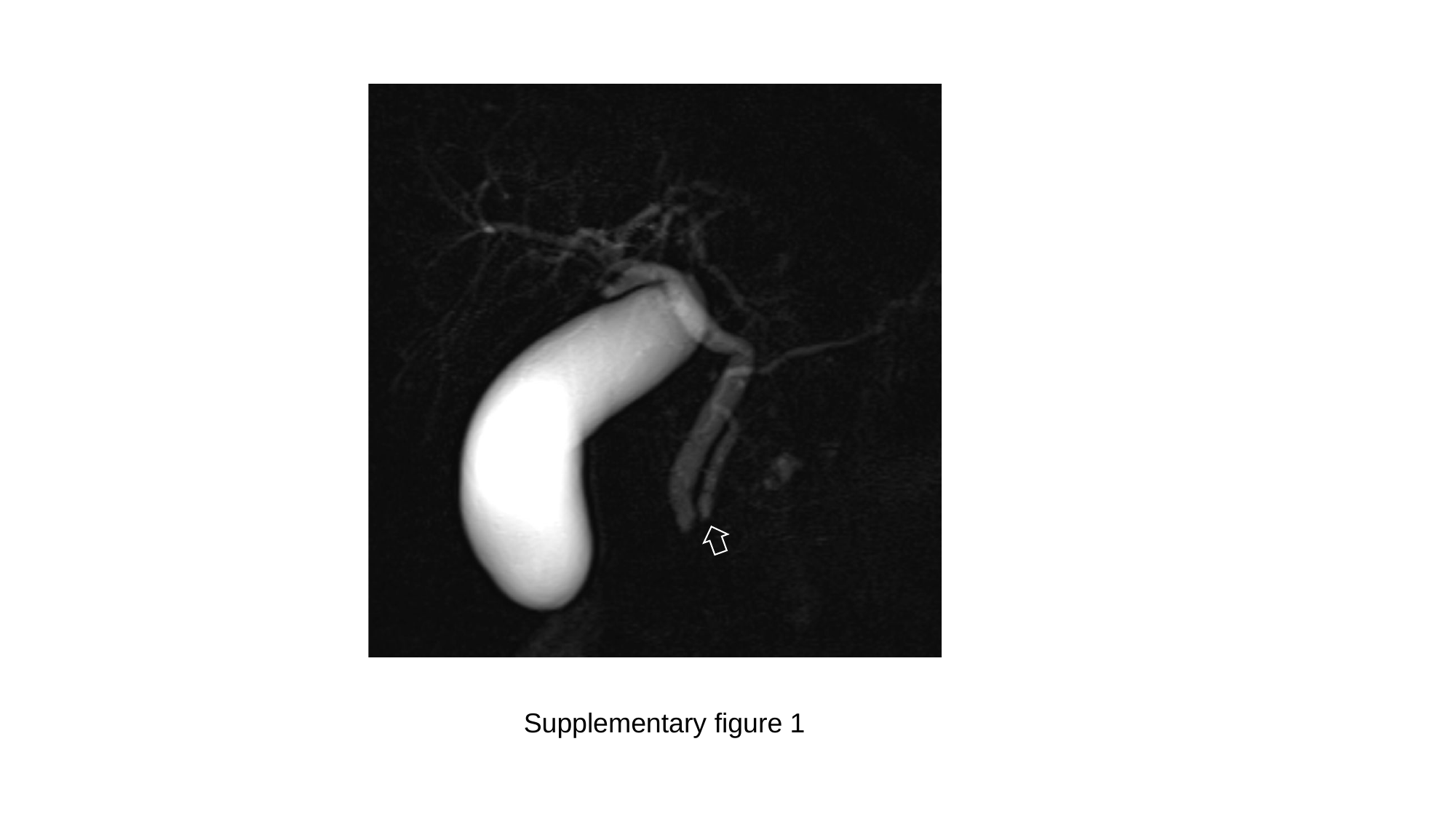

Supplementary figure 1

Supplement: Supplementary file 1 — MRCP image obtained on day 105 after discharge, which corresponds to day 75 after pancreatic stent removal: the previously noted focal cystic dilation of the pancreatic duct had resolved. [file DEO2-6-e70156-s002.pptx]
