# Supplementary material for: Wirsungocele as a Rare Cause of Recurrent Pancreatitis: Etiology and Therapeutic Insights
Source: DEN Open. 2025 Jun 5;6(1):e70156. doi: 10.1002/deo2.70156 (PMC12140840; doi:10.1002/deo2.70156)
Supplement: Supplementary file 2 — Video of the Endoscopy [file DEO2-6-e70156-s001.docx]

Supporting video can be downloaded here:

[Video of Endoscopy 1.27.mp4](https://wiley-my.sharepoint.com/:v:/p/yikegami/Ea7yRGEmCV9BrQ6INQrmkcIBY42_q_aTIbRx0V2VWimcow?e=dM3fK8)
